# Characterization of the immune cell landscape of patients with NAFLD

**DOI:** 10.1371/journal.pone.0230307

**Published:** 2020-03-13

**Authors:** Tom Diedrich, Silke Kummer, Antonio Galante, Andreas Drolz, Veronika Schlicker, Ansgar W. Lohse, Johannes Kluwe, Johanna Maria Eberhard, Julian Schulze zur Wiesch

**Affiliations:** Medical Department, University Medical Center Hamburg-Eppendorf, Hamburg, Germany; Medizinische Fakultat der RWTH Aachen, GERMANY

## Abstract

Multiple factors are involved in the pathogenesis of non-alcoholic fatty liver disease (NAFLD), but the exact immunological mechanisms that cause inflammation and fibrosis of the liver remain enigmatic. In this current study, cellular samples of a cohort of NAFLD patients (peripheral blood mononuclear cells (PBMC): n = 27, liver samples: n = 15) and healthy individuals (PBMC: n = 26, liver samples: n = 3) were analyzed using 16-color flow cytometry, and the frequency and phenotype of 23 immune cell subtypes was assessed. PBMC of NAFLD patients showed decreased frequencies of total CD3+, CD8+ T cells, CD56^dim^ NK cells and MAIT cells, but elevated frequencies of CD4+ T cells and Th2 cells compared to healthy controls. Intrahepatic lymphocytes (IHL) of NAFLD patients showed decreased frequencies of total T cells, total CD8^+^ T cells, Vd2^+^γδ T cells, and CD56^bright^ NK cells, but elevated frequencies of Vδ2-γδ T cells and CD56^dim^ NK cells compared to healthy controls. The activating receptor NKG2D was significantly less frequently expressed among iNKT cells, total NK cells and CD56^dim^ NK cells of PBMC of NAFLD patients compared to healthy controls. More strikingly, hepatic fibrosis as measured by fibroscan elastography negatively correlated with the intrahepatic frequency of total NK cells (r^2^ = 0,3737, p = 0,02). Hepatic steatosis as measured by controlled attenuation parameter (CAP) value negatively correlated with the frequency of circulating NKG2D^+^ iNKT cells (r^2^ = 0,3365, p = 0,0047). Our data provide an overview of the circulating and intrahepatic immune cell composition of NAFLD patients, and point towards a potential role of NK cells and iNKT cells for the regulation of hepatic fibrosis and steatosis in NAFLD.

## Introduction

Non-alcoholic fatty liver disease (NAFLD) is the most common liver disease in the western industrialized countries and is a growing burden to the public health systems with few approved therapeutic options currently available [1].

NAFLD encompasses two entities of different disease severity with (I) non-alcoholic fatty liver (NAFL) which is defined as mere hepatic steatosis without inflammation, and (II) non-alcoholic steatohepatitis (NASH) being defined by the presence of intrahepatic lobular inflammation and/or hepatocellular ballooning. A close relationship between NAFLD and metabolic syndrome (which clusters central obesity, dyslipidemia, insulin resistance, and arterial hypertension, has previously been highlighted [2]. Patients diagnosed with NASH, are at an increased risk of developing cirrhosis of the liver and hepatocellular carcinoma [3,4].

It is commonly thought that NAFLD pathogenesis occurs as a result of multiple processes taking place in parallel (rather than consecutively) and that may act additively, hence the term “multiple parallel hits hypothesis”. Possible pathogenic factors involved may include: insulin resistance, several genetic polymorphisms, microbial translocation and the effect of different lymphocyte populations [5].

In NAFLD patients, the stage of hepatic fibrosis is an important variable that correlates with relevant clinical outcomes such as liver transplantation and liver-related mortality [6–8].

The presence of lobular inflammation is an important risk factor for the development and progression of hepatic fibrosis [9].

Several immune cell types have been found to be increased and phenotypically distinct in the lobar inflammation observed in human and murine NAFLD compared to healthy liver. These cell types include: cytotoxic T cells, Th17 cells, regulatory T cells (Tregs), mucosal associated invariant T (MAIT) cells, γδT cells, iNKT cells, and natural killer (NK) cells [10]. However, the directionality of these observed differences is still controversially discussed, especially in the context of human NAFLD [10,11], and a comprehensive assessment of the peripheral and intrahepatic immune cell composition in human NAFLD is missing.

For a better understanding of the immunopathogenesis of human NAFLD and the development of novel immuno-therapeutic approaches, it will be important to elucidate immunological correlates of disease progression and the detailed cellular composition of peripheral blood and lobular inflammation in patients with NAFLD and their interplay with hepatic steatosis and fibrosis. For this purpose, we developed two comprehensive 16-color fluorescence-activated cell sorting (FACS) panels to characterize phenotype and functional capacity of T helper cells, cytotoxic T cells, Th17 cells, Tregs, MAIT cells, γδT cells, iNKT cells and NK cells, and tested it with cellular samples of patients of a “real world” clinical NAFLD cohort.

In this pilot study, we comprehensively assessed and visualized the peripheral and intrahepatic cellular immune landscape of patients at different stages of NAFLD. Our work is inspired by a description of the immune landscape in hepatocellular carcinoma (HCC) patients recently published by Rohr Udilova et al., and hints towards a possible role of iNKT and NK cells in NAFLD pathogenesis and regulation of hepatic steatosis as well as fibrosis [12].

## Materials and methods

### Study subjects

Written informed consent was obtained from all participants who were recruited for this study at the University Medical Center Hamburg-Eppendorf which was approved by the Ärztekammer Hamburg (PV4781, PV4081) and conducted in accordance with the declaration of Helsinki. The following clinical and demographic data were extracted by clinical chart review: age, sex, AST (asparate aminotransferase) levels, ALT (alanine aminotransferase) levels, GGT (gamma-glutamyl transferase) levels, liver elastography parameters such as liver elasticity and CAP (Controlled attenuation parameter) [13], grade of hepatic steatosis measured by ultrasound, the NAS score (NAFLD activity score) and the histopathological fibrosis score [14]. An overview of the clinical characteristics of the cohort is shown in **Table 1.**

**Table 1:** Cohort characteristics.

	HC PBMC	NAFLD PBMC	HL	NAFLD liver
**Subjects**	26	27	3	15
**Age, mean with SD (years)**	38	51,28 (σ = 13,86)	39 (σ = 6,245)	53,53 (σ = 12,2)
**Women (%)**	37	28	67	60
**AST, mean with SD (ULN)**	NA	0,955 (σ = 0,4262)	0,82 (σ = 0,3677)	1,641 (σ = 2,727)
**ALT, mean with SD (ULN)**	NA	1,378 (σ = 0,5723)	0,6935 (σ = 0,6414)	1,768 (σ = 0,8746)
**GGT, mean with SD (ULN)**	NA	2,752 (σ = 2,903)	1,552 (σ = 1,042)	6,79 (σ = 15,38)
**Platelet count, mean with SD (x10**^**9**^**/L)**	NA	206,5 (σ = 65,19)	295,5 (σ = 152,5)	242,9 (σ = 58,4)
**Fibroscan, mean with SD (kPa)**	NA	10,78 (σ = 15,47)	6,233 (σ = 2,706)	12,22 (σ = 8,258)
**CAP, mean with SD (dB/m)**	NA	315,7(σ = 79,69)	NA	256 (σ = 41,58)
**Steatosis sonography, median (IQR)**	NA	1,5 (1)	NA	NA
**Fibrosis score**^**1**^**, median (IQR)**	NA	NA	0 (0)	1,5 (2)
**NAS, median (IQR)**	NA	NA	0 (0)	4(1)

NA, not applicable. PBMC, Peripheral Blood Mononuclear Cell. HC, healthy control. NAFLD, non-alcoholic fatty liver disease. HL, healthy liver. AST, aspartate transaminase. ALT, alanine transaminase. GGT, Gamma glutamyl transpeptidase. CAP, controlled attenuation parameter. IQR, Interquartile range. NAS, NAFLD activity score. SD (σ), Standard deviation.

### Sample processing

Cryopreserved peripheral blood mononuclear cells (PBMC) and IHL were used after thawing for immunophenotypic staining as previously described by Dunay et al [15]. Liver biopsies were taken during mini-laparoscopy [16]. The biopsies were drawn into sterile Phosphate Buffered Saline (PBS) and intrahepatic lymphocytes (IHL) were directly processed as previously described by Eberhard et al [17]. After thawing the samples were stained and measured immediately.

### Immune phenotypic analysis of lymphocyte surface markers

Cells were stained using two different panels with Zombie NIR Fixable Viability stain (BioLegend, San Diego, USA) and combinations of the following fluorochrome-conjugated surface antibodies: CD4 (clone SK3), CD45 (clone HI30), CD56 (clone NCAM16.2), TCR-γ/δ (clone 11F2) all BD Biosciences, Heidelberg, Germany and CD8 (clone RPA-T8), HLA-DR (clone L243), CD45RA (clone HIT100), CD196 (CCR6) (clone G034E3), CD194 (CCR4 clone L29144), CD197 (CCR7) ((clone G043H7), CD57 (clone HNK-1), CD183 (CXCR3) (clone G025H7), CD38 (clone HIT2), CD161 (clone HP-3G10), CD25 (clone M-A251), CD3 (clone UCHT1), CD127 (clone A019D5), CD14 (clone HCD14), CD19 (clone HIB19), CD16 (clone 3G8), TCR Vα7.2 (clone 3C10), TCR Vα24-Jα18 (clone 6B11), CD314 (NKG2D) (clone 1D11), CD4 (clone SK3), TCR Vδ2-FITC, (clone B6), CD39-PE/Cy7 (clone A1) all BioLegend, San Diego, USA. Single-stained Comp Beads (Anti-Mouse Ig,κ/Negative Control Compensation Particles Set, BD Biosciences) were used for compensation. For live/dead compensation, Comp Beads stained with anti-CD14 (APC Cy-7, BioLegend) were applied. The exact composition of these two panels is displayed in **S1 Table**. All samples were run on a BD LSR Fortessa flow cytometer with FACS Diva version 8 (BD Biosciences) on a PC.

### Data analysis and statistics

Cytometric data were analyzed using FlowJo version 10.5.2 for Mac OS X (FlowJo, BD, Franklin Lakes, NJ, USA). Statistical analysis was performed using GraphPad Prism version 7.0c for Mac OS X (GraphPad Software, Inc., La Jolla, CA, USA). For statistical comparisons Mann-Whitney tests with an alpha value of 0.05 were performed. Pearson’s correlation was applied for bivariate correlation analysis. Data are expressed as means +/- standard deviation. Frequencies in the text are described as means unless stated otherwise. A p-value of less than 0.05 was considered significant.

## Results

### Study cohort

In this study, we present flow cytometric data of a cohort of NAFLD patients (PBMC: n = 27, liver samples: n = 15) in comparison with healthy individuals (PBMC: n = 26, liver samples: n = 3). PBMC of NAFLD patients were obtained at the outpatient clinic; a detailed overview of the cohort and respective subgroups is displayed in **S1 Fig**. The degree of fibrosis in patients for whom only PBMC were available, was solely determined by fibroscan (mean 10,78 kPa, range 2,7–75 kPa), whereas for patients of whom liver biopsies were available the result of the fibroscan (mean 12,22 kPa, range 4–30,7 kPa) and histological evaluation (median 1, range 1–4) were used. The degree of steatosis by CAP (mean 315,7 dB/m, range 150–328 dB/m) was either obtained by fibroscan elastography and the evaluation by conventional ultrasound (median 1), or it was assessed by the pathologist as part of the NAS (median 4, range 0–5). In the NAFLD cohort we defined NAFL as having a NAS <4 and NASH as having a NAS> 3 [14].

### Multicolor flow cytometry: Panel design and gating strategy

In order to comprehensively analyze the immunological cell signature of patients with NAFLD, we stained PBMC and IHL of NALFD patients and healthy volunteers using two separate multicolor FACS panels. Panel 1 was designed to assess the frequency and phenotype of different conventional T cell subsets including total T cells, cytotoxic T cells, T helper cells and Tregs). Panel 2 was designed to assess the frequency and phenotype of NK cells, as well as unconventional T cells such as iNKT cells, MAIT cells and γδ T cells. The composition of the two FACS panels is shown in **S1 Table**. Our standard gating strategy is depicted in **S2 Fig** with representative FACS plots of PBMC and IHL in **S3 Fig.** In short, after gating for singlets, CD45^+^, alive (Zombie NIR negative), CD14^-^ and CD19^-^ lymphocytes we then defined T cells via CD3 expression. T helper cells were gated as CD3^+^ and CD4^+^ and cytotoxic T cells as CD3^+^and CD8^+^. CD4^+^ T cell lineages were defined by excluding naïve cells (via exclusion of CD45RA^+^ cells) and expression of the following chemokine receptors: TH1 as CXCR3^+^and CCR6^-^, CCR4^-^; TH2 as CCR4^+^and CCR6^-^, CXCR3^-^ and TH17 as CCR6^+^, CCR4^+^, CD161^+^and CXCR3^-^. CD4^+^ Tregs were defined by surface marker expression as CD25^+^ and CD127^-^. Invariant Natural killer T cells (iNKT) were defined as TCRVα24Jα18^+^ and CD3^+^, γδT cells were defined as pan γδ TCR^+^ with a subdivision in Vδ2^+^ γδT cells and Vδ2^-^ γδT cells, MAIT cells were defined as CD3^+^, CD4^-^, CD161^+^, TCR Vα7.2^+^. NK cells were definded as CD16 and CD56^+^. Based on the differentiation markers CD45RA and CCR7, we defined naïve and memory subsets on T cells (CCR7^−^ CD45RA^+^ terminal effector-T_EMRA_; CCR7^+^/CD45RA^+^ naïve T cells-T_naïve_; CCR7^−^ CD45RA-–effector memory–T_EM_; CCR7^+^ CD45RA^-^–central memory–T_CM_). Accordingly, the FACS panel 1 was designed to assess the activation, senescence and differentiation status (T_naïve_, T_CM_, T_EMRA_, T_EM)_ of different T cell subsets, namely: cytotoxic T cells, TH1 cells, TH2 cells, TH17 cells, regulatory T cells. Panel 2 was designed to assess additional T cell and lymphocyte subsets with emphasis on different unconventional T cell substes: iNKT cells, γδT cells with a subdivision in Vδ2^+^ γδT cells and Vδ2^-^ γδT cells, MAIT cells as well as NK cells with their subdivision in CD56^bright^ NK cells and CD56^dim^ NK cells (**S1 Table**).

### Immune cell patterns of PBMC and IHL of NAFLD patients

**Fig 1:**
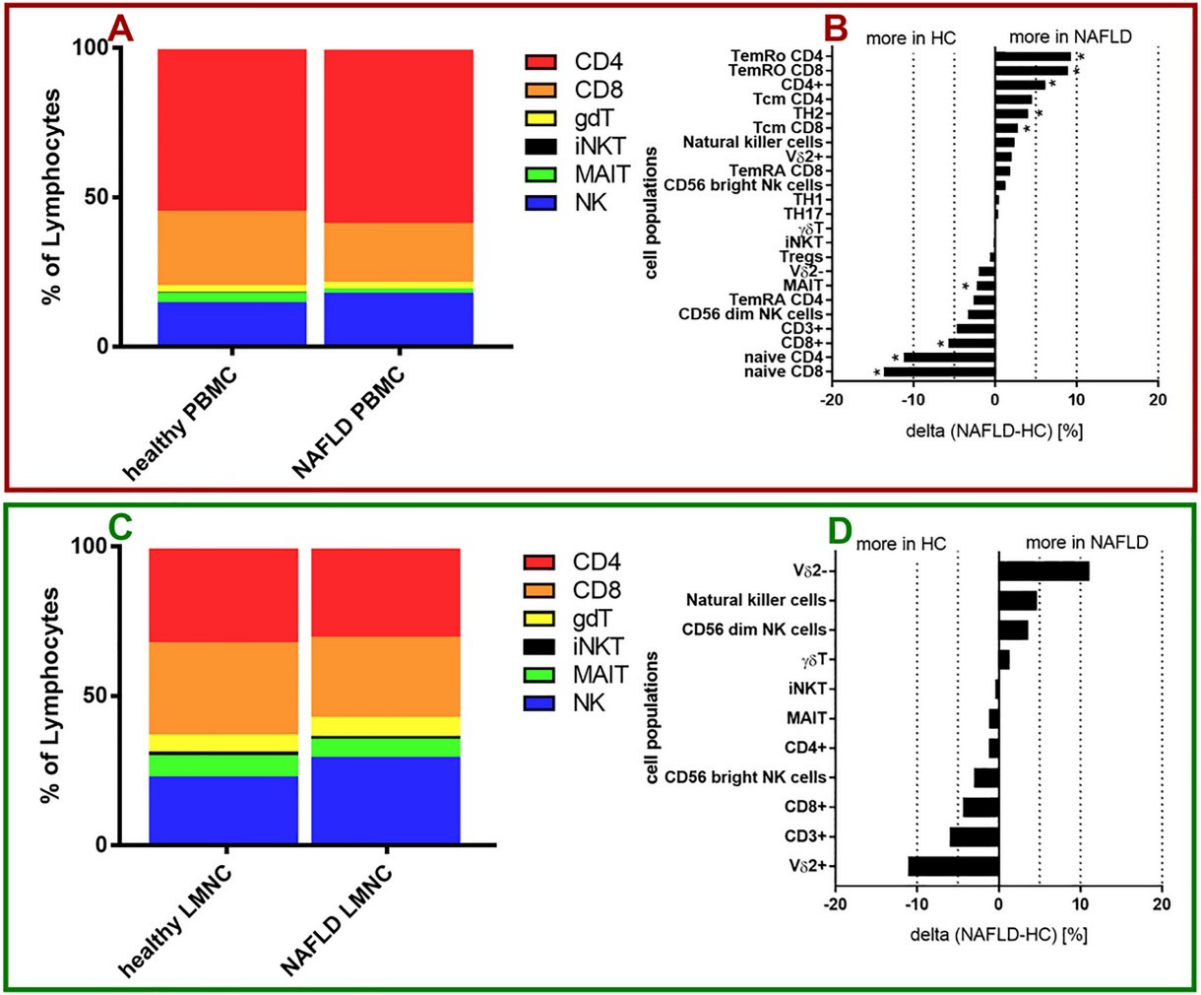
Immune cell composition in NAFLD. (A, C) Composition of infiltrating immune cells in PBMC (A) and liver samples (C) of healthy controls as well as NAFLD patients summarized from calculated mean values of each patient group. (B, D) Changes of immune cell composition between NAFLD patients and healthy controls in PBMC samples (B) and liver samples (D). (red Box) PBMC data. (green Box) liver data. (B,D) For comparisons Mann-Whitney test was used. *p<0.05.

In PBMCs of NAFLD patients, the following populations showed increased frequencies in comparison to healthy controls: TemRO CD4^+^ T cells (28,32% (standard deviation (SD) = 11,97)) vs. 19,03% (SD = 8,018), p = 0,0107), TemRO CD8^+^ T cells (25,22% (SD = 10,37) vs. 16,27% (SD = 9,624), p = 0,0068), total CD4^+^ T cells (67,93%(SD = 11,83) vs. 59,9%(SD = 7,32), p = 0,0097), T central memory CD4^+^ T (29,72%(SD = 9,089) vs. 25,2% (SD = 14,03), p = 0,0633), TH2 cells (18,41% (SD = 6,788) vs. 14,36% (SD = 6,387), p = 0,0417) and central memory CD8^+^ T cells (7,06% (SD = 4,91) vs. 4,261% (SD = 4,123), p = 0,0273). In contrast, PBMC samples of NAFLD patients showed reduced frequencies of the following populations compared to healthy controls: naïve CD8^+^ T cells (29,59% (SD = 15,7) vs. 43,22% (SD = 17,5), p = 0,0062), naïve CD4^+^ T cells (38,39% (SD = 14,42) vs. 49,58% (SD = 13,84), p = 0,0060), total CD8+ T cells (26,12% (SD = 11,33) vs. 32,47% (SD = 6,432), p = 0,0173), total CD3^+^ T cells (78,22% (SD = 12,76) vs. 82,92% (SD = 5,794), p = 0,2744), CD56^dim^ NK cells (84,01% (SD = 8,775) vs. 87,33% (SD = 6,444), p = 0,2605), TemRA CD4^+^ T cells (3,579% (SD = 2,54) vs. 6,202% (SD = 11,23), p = 0,2698) and MAIT cells (1,841%(SD = 1,738) vs. 4,073% (SD = 3,44), p = 0,0007) (**S2 Table**). NK cells, CD56^dim^ NK cells, Vδ2^+^ γδT cells, Vδ2^-^ γδT cells, TH1 cells, TH17 cells, Tregs and iNKT cells had similar frequencies in PBMC of NAFLD patients compared to healthy controls (**Fig 1A and 1B**). Of note, in PBMC of both healthy individuals and NAFLD patients almost 80% of the total number of lymphocytes were T cells.

The intrahepatic cellular composition slightly differed from the peripheral composition in the blood as described above (**Fig 1A and 1C**) with a decreased proportion of T cells in NAFLD patients compared to healthy controls. Additionally, in liver samples Vδ2^-^ γδT cells, NK cells and CD56^dim^ NK cells were detected at higher frequencies in NAFLD patients; whereas Vδ2^+^ γδT cells, total T cells, total CD8^+^ T cells and CD56^bright^ NK cells were detected at slightly lower frequencies compared to healthy controls (**S3 Table**). However, these differences were not statistically significant. γδT cells, iNKT cells, MAIT cells, and CD4^+^ T cells were present at similar frequencies in liver samples of NAFLD patients compared to healthy controls. NK cells composed a much bigger portion of total lymphocytes in liver samples compared to peripheral blood with 29,25% (SD = 14,37) versus 17,07% (SD = 10,12) for NAFLD and 24,57% (SD = 13,85) versus 14,68% (SD = 5,523) for healthy controls (**Fig 1C and 1D**). Rah et al. previously demonstrated that a knockout of CD38 of NK cells is associated with reduced cytotoxicity using mouse models targeting different steps in the signal cascade of CD38 on NK cells [18]. Interestingly, we found significantly higher frequencies of CD38^+^ NK cells (73,00% versus 56,18%, p = 0,0181) and CD38^+^ CD56^dim^ NK cells (74,87% versus 56,68%, p = 0,0136) in PBMC of NAFLD patients compared to healthy controls (**S4B Fig**). We did not measure CD38 expression of intrahepatic immune cells due to the paucity of cells that only lowed for running panel 1. With regard to CD39 and CD57, we found only minor differences of the expression on all peripheral and intrahepatic immune cell populations examined (**S4C, S4D, S5B and S5D Figs**).

In the initial analysis, we compared NAFLD patients with healthy controls to get an overview of the cellular composition. However, the intrahepatic analysis was particularly limited by the small number of healthy controls.

However, in order to validate our findings and to determine which immune cell populations play a role in NAFLD pathogenesis we then focused on the analysis of cell populations in samples from NAFLD patients and correlated frequencies with disease-related clinical markers. Immune cell frequencies of patients with NAFLD were correlated with hepatic steatosis (measured by CAP), as a disease defining clinical marker and hepatic fibrosis (measured by fibroscan) which correlates with relevant clinical outcomes such as liver transplantation and liver-related mortality [6–8].

### NKG2D^+^NK cell and CD127^+^T cell frequencies correlate with the degree of hepatic fibrosis of NAFLD patients

NK cells can contribute to a pro-inflammatory environment through the production of cytokines such as interferon-γ (IFN-γ) and tumor necrosis factor (TNF) [19] and are enriched in the liver where they comprise up to 30% of all lymphocytes [11].

It has been shown that NK cells are able to kill hepatic stellate cells (HSC) and thus protect against hepatic fibrosis in murine and human models [20].

**Fig 2:**
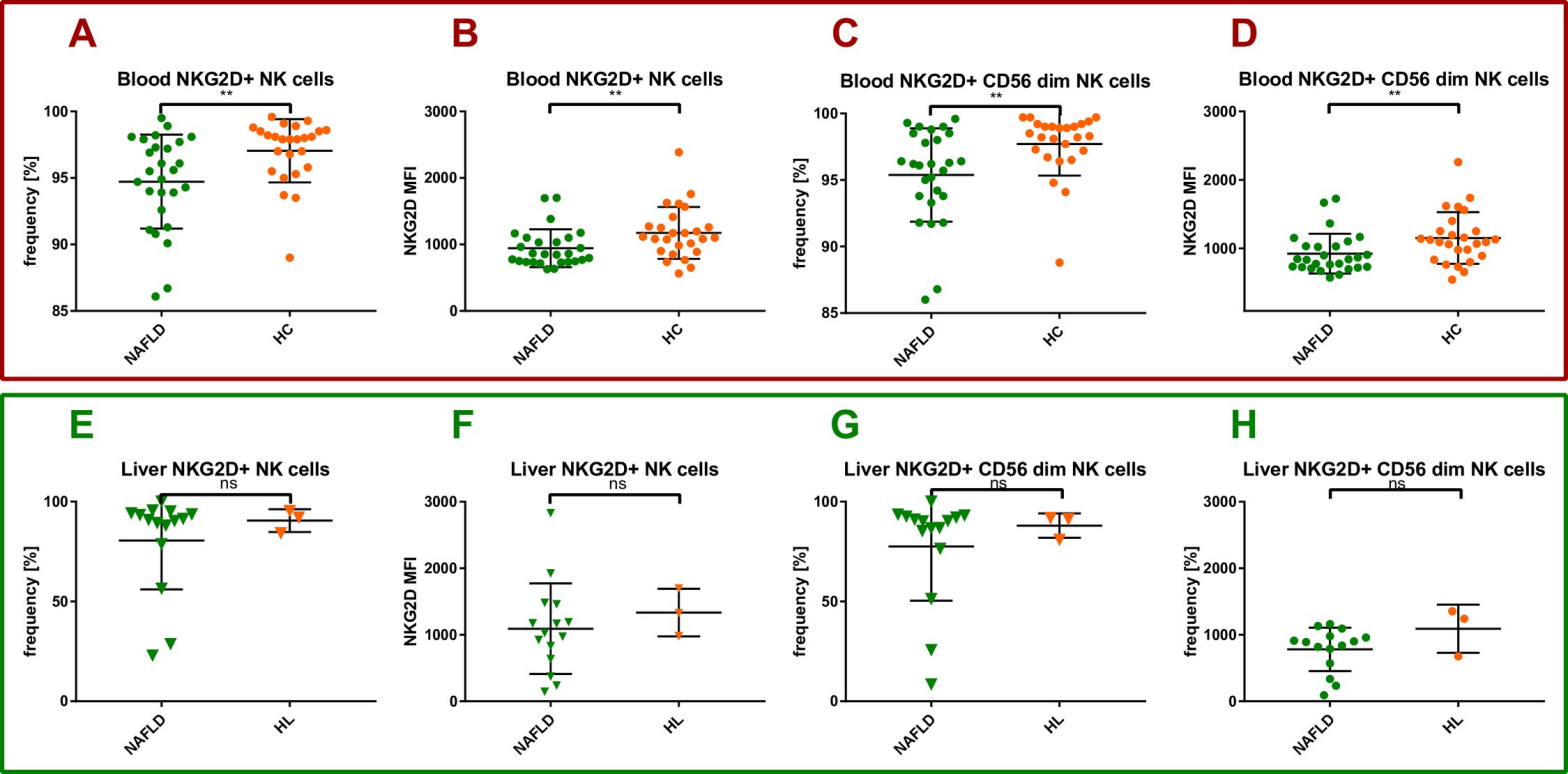
Peripheral and intrahepatic NKG2D positive NK cells in NAFLD. Healthy PBMC controls (HC), healthy liver controls (HL), NAFLD liver and PBMC samples (NAFLD), mean fluorescence intensity (MFI). Each spot represents one patient. (A-D) PBMC data. (E-H) intrahepatic data. (A) NKG2D+NK cell frequency in PBMCs of NAFLD patients compared to healthy controls. (B) NKG2D MFI of NK cells in PBMCs of NAFLD patients compared to healthy controls. (C) NKG2D+CD56dim NK cell frequency in PBMCs of NAFLD patients compared to healthy controls. (D) NKG2D MFI of CD56^dim^ NK cells in PBMCs of NAFLD patients compared to healthy controls. (E) NKG2D+ NK cell frequency in liver samples of NAFLD patients compared to healthy controls. (F) NKG2D MFI of NK cells in IHLs of NAFLD patients compared to healthy controls. (G) NKG2D+ CD56dim NK cell frequency in liver samples of NAFLD patients compared to healthy controls. (H) NKG2D MFI of CD56^dim^ NK cells in IHLs of NAFLD patients compared to healthy controls. For Comparisons Mann-Whitney test was used. *p<0.05, **p>0.01.

Additionally liver NK cells have the ability to kill activated hepatic stellate cells in the fibrotic liver via NKG2D signaling as reported in murine models of hepatic fibrosis [21]. Our results show that the frequency of peripheral blood NKG2D^+^ NK cells was significantly lower in NAFLD patients compared to healthy controls (94,72% (SD = 3,529) vs. 97,04% (SD = 2,375), p = 0,0062) (**Fig 2A**). The same held true for the frequency of peripheral blood NKG2D^+^ CD56^dim^ NK cells (95,38% (SD = 3,507) vs. 97,7% (SD = 2,364), p = 0,0021) (**Fig 2C**). Likewise, the MFI of NKG2D on peripheral blood NK cells and CD56^dim^ NK cells was significantly lower in NAFLD patients compared to healthy controls (943,5 (SD = 285,1) vs. 1173 (SD = 389,9), p = 0,0087 and 923,0 (SD = 291,0) vs. 1151 (SD = 378,2), p = 0,0097) (**Fig 2B and 2D).** Similarly, in the liver, the frequency of NKG2D^+^ total and CD56^dim^ NK cells showed the same trend in NAFLD patients compared to healthy controls (80,55% (SD = 24,49) vs. 90,5% (SD = 5,7) and 77,52% (SD = 27,16) vs. 87,97% (SD = 6,127), respectively (**Fig 2E and 2G**). In addition, the MFI of NKG2D on intrahepatic NK cells and CD56^dim^ NK cells was lower in NAFLD patients compared to healthy controls (1092 (SD = 681,0) vs. 1334 (SD = 357,1) and 780,2 (SD = 327,3) vs. 1091 (SD = 362,5)) (**Fig 2F and 2H**). However, these differences did not reach statistical significance.

**Fig 3:**
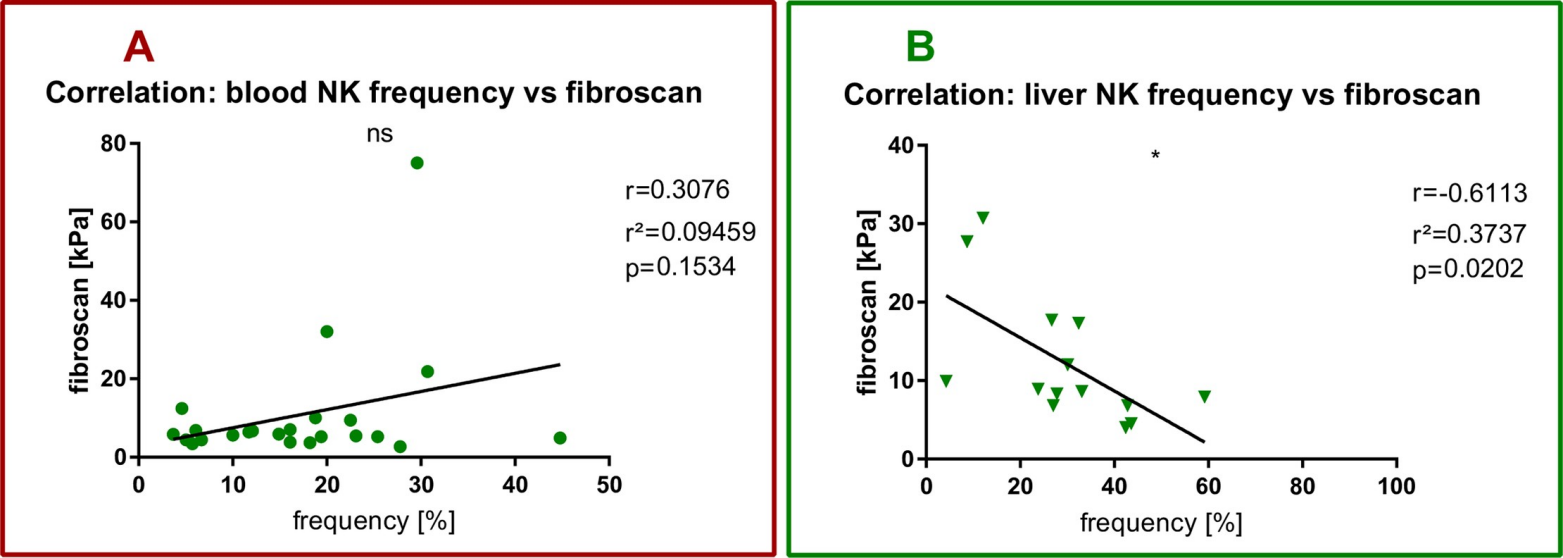
NK cells and hepatic fibrosis in NAFLD. Each spot represents one patient. (A) PBMC data. (B) intrahepatic data. (A) Pearson’s correlation between peripheral NK cell frequency and fibroscan with Pearsons r, r^2^ and p value. (B) Pearson’s correlation between intrahepatic NK cell frequency and fibroscan with Pearsons r, r^2^ and p value. *p<0.05, **p>0.01.

We found a statistically significant negative correlation between the intrahepatic NK cell frequency and fibroscan liver elastography (Pearson r^2^ = 0,3737, p = 0,0202) (**Fig 3B**) which could not be observed in the peripheral blood (Pearson r^2^ = 0,09459, p = 0,1534) (**Fig 3A**).

**Fig 4:**
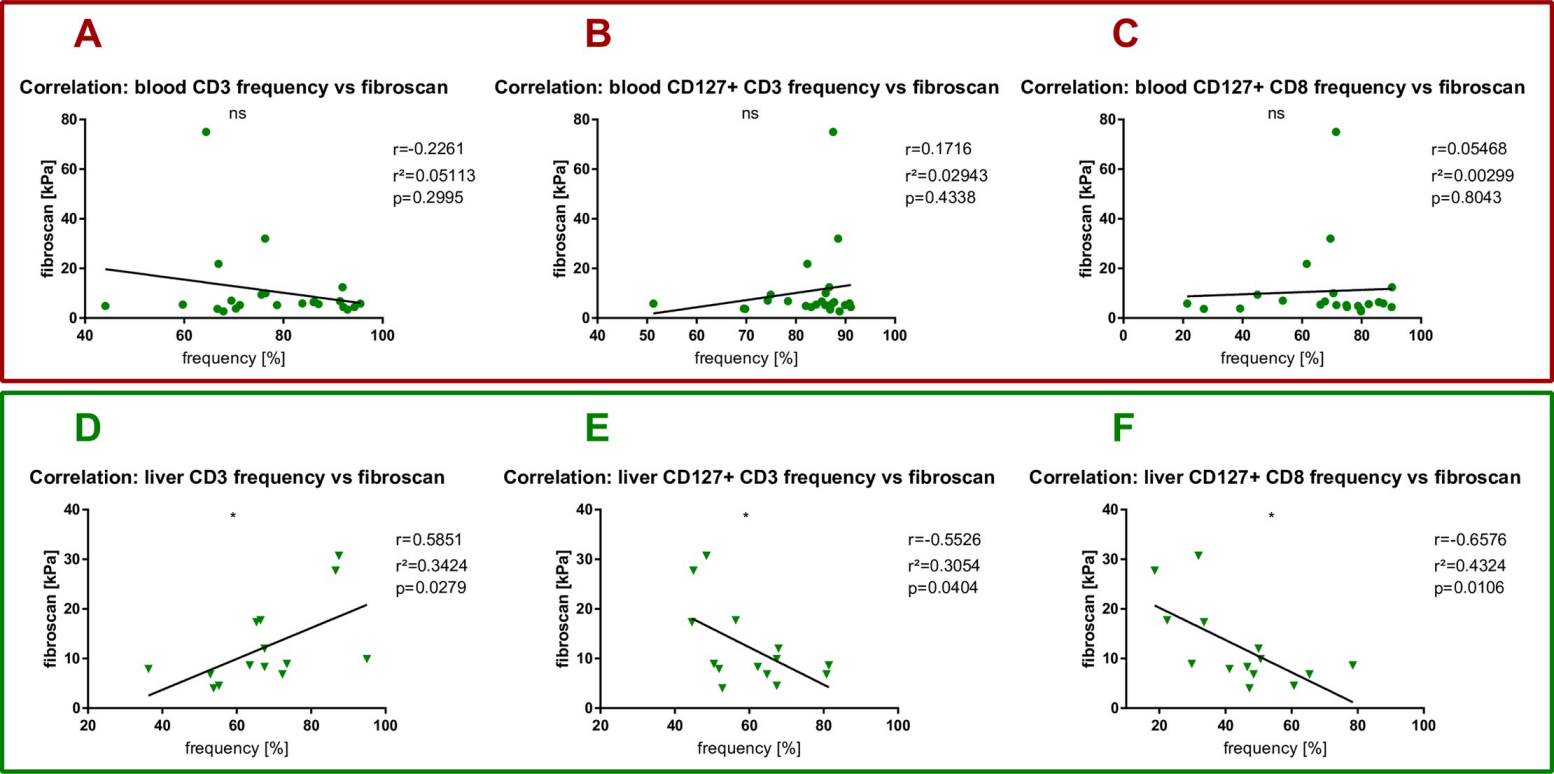
T cells and hepatic fibrosis in NAFLD. Each spot represents one patient. (A-C) PBMC data. (D-F) intrahepatic data. (A) Pearson’s correlation between peripheral T cell frequency and fibroscan with Pearsons r, r^2^ and p value. (B) Pearson’s correlation between peripheral CD127 positive T cell frequency and fibroscan with Pearsons r, r^2^ and p value. (C) Pearson’s correlation between peripheral CD127 positive CD8 positive T cell frequency and fibroscan with Pearsons r, r^2^ and p value. (D) Pearson’s correlation between intrahepatic T cell frequency and fibroscan with Pearsons r, r^2^ and p value. (E) Pearson’s correlation between intrahepatic CD127 positive T cell frequency and fibroscan with Pearsons r, r^2^ and p value. (F) Pearson’s correlation between intrahepatic CD127 positive CD8 positive T cell frequency and fibroscan with Pearsons r, r^2^ and p value. *p<0.05.

Moreover, the total frequency of intrahepatic CD3^+^ T cells positively correlated with the degree of fibrosis as measured by fibroscan (r^2^ = 0,3424, p = 0,0279) (**Fig 4D**). Again, no correlation between the two parameters could be observed in the peripheral blood (Pearson r^2^ = 0,05113, p = 0,2995) (**Fig 4A**). In contrast to the positive correlation of intrahepatic total T cells, we detected a statistically significant negative correlation between intrahepatic CD127^+^ T cells and CD127^+^CD8^+^T cells and fibroscan (r^2^ = 0,3054, p = 0,0404 and r^2^ = 0,6576, p = 0,0106) (**Fig 4E and 4F)** that was not detectable in peripheral blood (**Fig 4B and 4C**). These correlations point towards a potential involvement of CD127^+^ T cells and CD127^+^ cytotoxic T cells for the regulation of hepatic fibrosis.

### The frequencies of NKG2D^+^ iNKT cells and CD127^+^ NK cells correlate with the degree of hepatic steatosis

Unlike conventional T cells which require peptide presentation via the MHC, iNKT cells exclusively recognize lipid antigens presented by the MHC-like molecule CD1d. iNKT cells also express an invariant TCRα chain (Vα24Jα18) which is characterized by a very limited selection of TCRβ proteins. Even though it is now widely accepted that NKT cells are depleted in murine-models of liver steatosis, the exact involvement of NKT cells in human NAFLD is somewhat controversial and there have been reports of both an increase but also a decrease in liver iNKT cell numbers when comparing healthy controls to NASH and NAFL patients [10].

**Fig 5:**
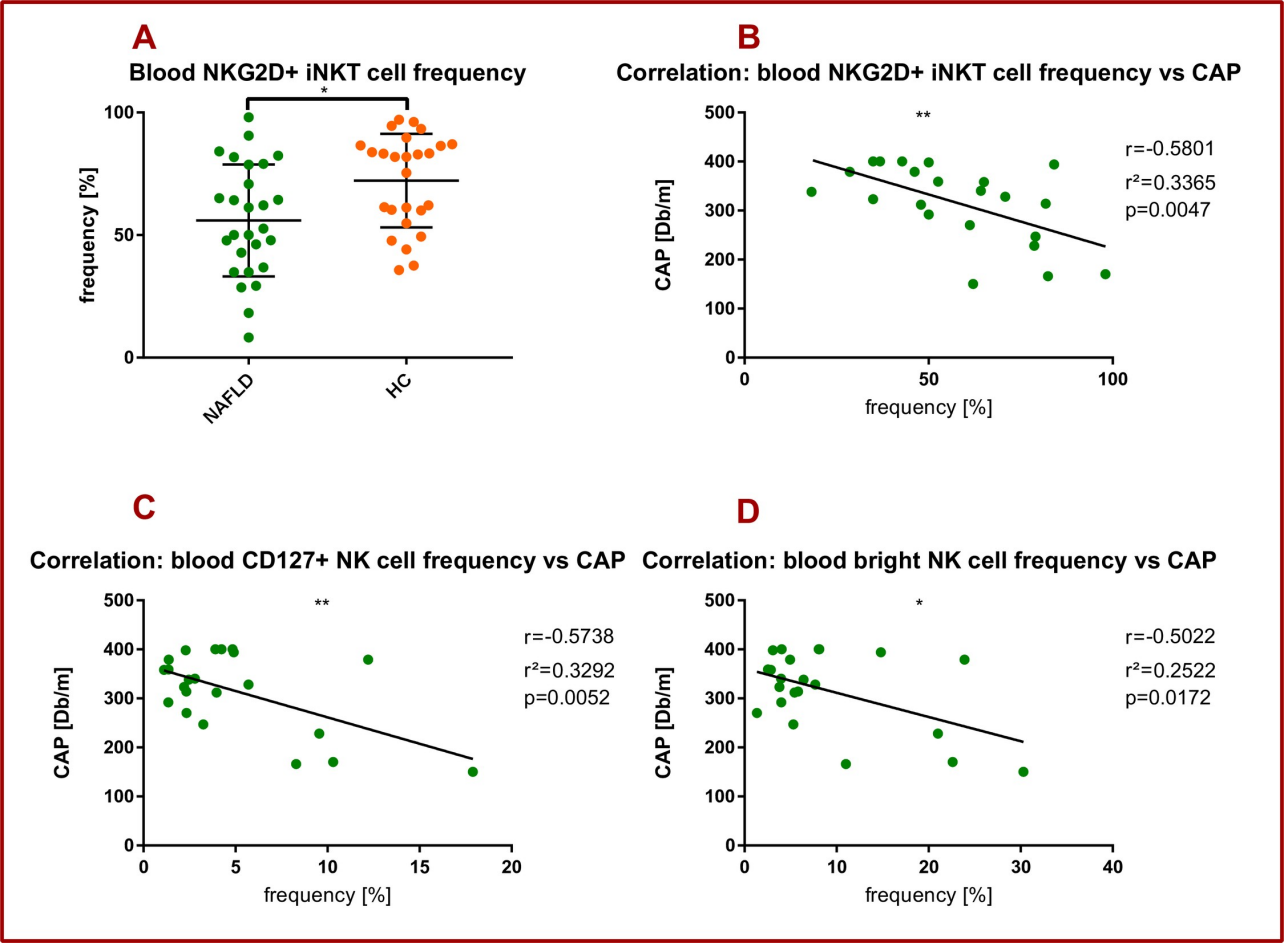
NKG2D^+^ iNKT cells and hepatic steatosis in NAFLD. Healthy PBMC controls (HC), healthy liver controls (HL), NAFLD liver and PBMC samples (NAFLD). Each spot represents one patient. (A-D) PBMC data. (A) NKG2D^+^ iNKT cell frequency in PBMCs of NAFLD patients compared to healthy controls. For comparison Mann-Whitney test was used. (B) Pearson’s correlation between peripheral NKG2D^+^ iNKT cell frequency and CAP with Pearsons r, r^2^ and p value. (C) Pearson’s correlation between peripheral CD127^+^ NK cell frequency and CAP with Pearsons r, r^2^ and p value. (D) Pearson’s correlation between peripheral CD127^+^ CD56 bright NK cell frequency and CAP with Pearsons r, r^2^ and p value. *p<0.05, **p>0.01.

In the current study, the frequency of peripheral blood NKG2D^+^ iNKT cells was significantly lower in NAFLD patients compared to healthy individuals (55,94% (SD = 22,85) vs. 72,2% (SD = 19,11), p = 0,0103) (**Fig 5A**). Moreover, we found a statistically significant negative correlation between the peripheral (but not intrahepatic) NKG2D+ iNKT frequency and the CAP value (r^2^ = 0,3365 p = 0,0047) (**Fig 5B**). We found a statistically significant negative correlation between peripheral blood CD127+ NK cells and CAP (r^2^ = 0,3292, p = 0,0052) (**Fig 5C**). The same held true for peripheral blood CD127+ CD56dim NK cells and the CAP value (r^2^ = 0,3292, p = 0,0052) (**Fig 5D**).

## Discussion

One aim of this explorative clinical immunology study was to devise practical and easy to use multicolor FACS panels to study the frequency and phenotype of a broad range of peripheral and intrahepatic lymphocyte subpopulations in heathy individuals and NAFLD patients. Here, we report on ranges and possible shifts that can be expected in specific lymphocyte subpopulations in patients with NASH versus healthy controls as reference for further human immunological NAFLD studies (**Fig 1A–1D**). Of particular interest for further investigation were those cell populations with frequency alterations, that correlated with disease-relevant markers such as hepatic steatosis or hepatic fibrosis (**Figs 2–5**).

We found a significant correlation between intrahepatic T cells and higher fibrosis stages (as measured via fibroscan) in NAFLD patients (**Fig 4D**). The general presence of lobular inflammation is an important risk factor for the occurrence of hepatic fibrosis [9]. Of note, this immunological signature was unique for the intrahepatic compartment and we did not find any correlation between peripheral T cell frequency and hepatic fibrosis (**Fig 4A**).

It has been proposed that IL-7 and its receptor might play a role in the development of NAFLD. On the one hand, it has been shown that serum IL-7 levels were negatively associated with fibrosis in NAFLD [22]. On the other hand, CD127 expression is controlled by an IL-7-induced downregulation [23]. We therefore hypothesized that there were positive correlations between the frequencies of CD127^+^ T cells and CD127^+^ CD8 cells and the fibroscan value in NAFLD patients.

Interestingly, we found a strong correlation between liver fibrosis and loss of the expression the alpha chain of the IL-7 receptor (CD127^+^) of total intrahepatic (but not peripheral) CD3+ and CD8^+^ T cells (**Fig 4E**). While the significance of this finding is unclear at this timepoint and detailed T cell subset analysis is not available, we suggest that the finding merits further follow-up studies. In general, down-regulation of CD127 on T cells could be a sign of increased intrahepatic inflammation, homing of activated T cells and loss of T cell homeostasis [23]. It is intriguing in this respect that toll-like receptor mediated increased IL-7 production by hepatocytes upon LPS exposure has been described [24]. Future studies should optimally integrate obtained T cell data with measurement of circulating markers of inflammation, microbial translocation and relevant cytokines.

On the other end of the spectrum of descriptive observations concerning CD127, we detected a signal involving CD127^+^NK cells which have also not yet been described in the context of NAFLD. Gasteiger et al described murine CD127^+^ NK cells as immature cells with a high potential for self-renewal and producing increased amounts of TNF and IFN-γ upon stimulation with IL-12 and IL-18 [25]. We found a statistically significant negative correlation between peripheral blood CD127^+^ NK cells and the CAP value. CAP is a marker that can be considered as a surrogate marker for hepatic steatosis [13]. The same held true for peripheral blood CD127^+^ CD56^bright^ NK cells and the CAP value. This is in line with the finding that NK cells are involved in protective immune responses directed against liver fibrosis and hepatocellular carcinoma [26]. This correlation between CD127^+^ NK cells and the CAP value points towards a potential role of CD127^+^ NK cells for the regulation of hepatic steatosis.

In murine models of hepatic fibrosis liver NK cells have the capability to kill activated hepatic stellate cells in fibrotic liver via NKG2D signaling as reported in murine models of hepatic fibrosis [21]. Recently, Stiglund et al. found higher MFI of NKG2D on circulating NK cells of NASH patients compared with NAFL patients, but no correlation with hepatic fibrosis was found [11]. We report a significant negative correlation between intrahepatic NK cell frequency and fibrosis measured by fibroscan (**Fig 3B**). This correlation could indicate a possible association between NK cell function and human hepatic fibrosis in NAFLD. We measured significantly lower frequencies of NKG2D^+^ total and CD56^dim^ NK cells as well as significantly lower MFI of NKG2D on total NK cells and CD56^dim^ NK cells in PBMC of NAFLD patients compared to healthy individuals (**Fig 2A and 2C**). A similar statistical trend was observed for intrahepatic NK cells. These data might indicate a profibrotic role of NK cells due to a lack of NKG2D^+^NK cells and lower levels of NKG2D on NK cells. One limitation of our efforts was the limited availability of liver samples.

The role of iNKT cells in the development and regulation of NAFLD is somewhat controversial [10]. On the one hand multiple studies have shown an attenuating influence of iNKT cells on various aspects of NALFD [27–30], on the other hand studies have shown no or even contrary effects [29,31–35]. Kuylenstierna et al. described three mechanisms by which NKG2D exerts an influence on the function of human NKT cells. First, NKG2D^+^ NKT cells expressed perforin with orientation towards NKG2D-ligand-expressing target cells. Second, NKG2D engagement led to degranulation and target cell killing. Third NKG2D engagement enhanced TCR-mediated NKT activation [36]. In our study the frequency of peripheral blood NKG2D^+^ iNKT cells was significantly lower in NAFLD patients compared to healthy controls (**Fig 5A**). Interestingly, we found a significant negative correlation between the frequency of peripheral blood NKG2D^+^iNKT cells and the CAP value. As seen on NK cells this suggests a beneficial role of NKG2D expression on iNKT cells, having potentially a negative regulatory impact on hepatic steatosis.

One major limitation of our study was the small number of healthy liver samples available for analysis. A larger sample size of healthy liver samples in future studies is needed to detect significant differences. Another limitation was that since lobular inflammation is a defining criterion for NASH, but not NAFL, patients in our NAFLD cohorts may have been too healthy to measure relevant aspects related to lobular inflammation.

The role of Treg and TH17 cells in NAFLD is still not clear and is complicated by the considerable differences in the definition of these cell populations and the methodology and cohorts used in different studies. Rau et al. found significantly higher frequencies of intrahepatic TH17 cells in NASH compared with NAFL patients. However, that pattern was not observed in the peripheral blood of NASH patients compared to NAFL patients [37]. Similarly, Vonghia et al. found no difference of the IL-17 plasma concentration of NASH patients compared to healthy controls [38]. In our study, we also did not find any differences in the frequency of TH17 cells in the peripheral blood of NAFLD patients compared to healthy controls (**Fig 1B, S4B Fig**). However, it has been shown that IL-17 can have profibrotic effects by activation of hepatic stellate cells (HSC) [39]. Furthermore, multiple experimental murine and in vitro models have reported that IL-17 administration can lead to an increase in hepatic steatosis [10]. Considering these diverging findings regarding the role of TH17 cells and IL-17 in the human NAFLD context, further studies are needed to shed more light on their role. Adding to the available information, Vonghia et al. reported a shift in the Treg TH17 cell balance in NASH patients compared to obese non NASH patients assessed by a decreased IL-10/IL-17 ratio [38]. Rau et al described a similar shift in liver samples of NASH patients compared to NAFL patients assessed by TH17/Treg ratio [37]. On the contrary, Bertola et al detected a shift towards the opposite direction in liver samples of NASH patients compared to S3 obese patients and obese S0 patients assessed by an increase in IL-10/IFNγ ratio [40]. Söderberg et al. reported increased levels of Tregs in liver samples of NASH patients compared to non-NASH patients [41]. In our study, we found no differences in the frequency of Tregs in the peripheral blood of NAFLD patients compared to healthy controls (**Fig 1B, S4B Fig**). Unfortunately, due to low IHL cell numbers in each liver biopsy, FACS panel 2 was not stained and intrahepatic changes of Treg and TH17 cell frequencies could not be assessed.

NAFLD is a highly complex disease, with multiple pathogenic factors and also extrahepatic manifestations. Van Herck et al has previously summarized immune cell alteration in subcutaneous fat tissue, visceral fat tissue, liver and blood of humans and mice [10]. However, there is a lack of data regarding the interplay of components of the immune between these different tissues. Thus, future studies should aim for larger, well-characterized cohorts with samples from all the above-mentioned tissues in parallel with analysis of soluble inflammation markers and microbial metabolites [42]. Atherosclerosis, hypertension, ill-controlled diabetes mellitus type II and adipose tissue inflammation are common comorbidities in NAFLD patients and thus potential confounding factors that should be controlled for [2].

In summary, using two custom-designed Flow FACS panels we were able to provide an extensive characterization of the peripheral and intrahepatic immune cell composition in NAFLD patients. Our data hint towards a potential role of NK cells and iNKT cells for the regulation of hepatic fibrosis and steatosis in NAFLD warranting future functional and longitudinal studies focusing on these cell populations in NAFLD.

## Supporting information

S1 Fig Absolute and relative sample size of cohorts. Samples of patients with Non-alcoholic fatty liver disease (NAFLD), healthy controls (healthy), patients with non-alcohloic fatty liver (NAFL) and non-alcoholic steatohepatitis (NASH).
(TIF)

S2 Fig Standard gating strategy. Standard gating strategy was performed on singlet, CD45+, live, CD14-, CD19- lymphocytes. Unconventional T cells and NK cells definition: NKT (TCRVα24Jα18), MAIT (CD161, TCRVα7.2), γδT cells (panγδ, TCRVδ2), NK (CD16, CD56). Conventional T cells definition: T helper cells (CD4+), cytotoxic T cells (CD8), TH1 (CCR6-, CCR4+, CXCR3-), TH2 (CCR6-, CCR4-, CXCR3+), TH17 (CCR6+, CCR4+, CXCR3-, CD161+) as displayed above.
(TIF)

S3 Fig Representative FACS plots of (A) PBMC samples and (B) intrahepatic samples. Shown are CD3^+^ T cells of lymphocytes, CD4^+^ and CD8^+^ T cells of CD3^+^ T cells and CD127^+^, CD39^+^ and NKG2D^+^ cells of CD3^+^ T cells. In PBMC samples also CD38^+^ and CD57^+^ CD3+ T cells were analyzed.
(TIF)

S4 Fig Peripheral blood differences of immune cell frequencies in NAFLD. (A-F) Quantified relative differences of immune cell composition between NAFLD patients and healthy controls in PBMC samples. *p<0.05.
(TIF)

S5 Fig Intrahepatic differences of immune cell frequencies in NAFLD part 1. (A,B) Quantified relative differences of immune cell composition between NAFLD patients and healthy controls in liver samples. (C,D) Quantified relative differences of immune cell composition between NASH patients and NAFL patients in liver samples. *p<0.05.
(TIF)

S6 Fig Intrahepatic differences of immune cell frequencies in NAFLD part 2. (A,B) Quantified relative differences of immune cell composition between NAFLD patients and healthy controls in liver samples. (C,D) Quantified relative differences of immune cell composition between NASH patients and NAFL patients in liver samples. *p<0.05.
(TIF)

S1 Table FACS panel composition. (DOCX)

S2 Table Immune cell frequencies in PBMC of NAFLD patients and healthy controls. PBMC, Peripheral Blood Mononuclear Cell. HC, healthy control. NAFLD, non-alcoholic fatty liver disease. HL, healthy liver. Mean immune cell frequencies with standard deviations of PBMC of NALFD patients and healthy controls. p values were calculated with Mann-Whitney test.
(DOCX)

S3 Table Immune cell frequencies in IHL of NAFLD patients and healthy controls. IHL, intrahepatic lymphocyte. NAFLD, non-alcoholic fatty liver disease. HL, healthy liver. Mean immune cell frequencies with standard deviations of IHL of NALFD patients and healthy controls. p values were calculated with Mann-Whitney test.
(DOCX)
