# Supplementary figures and images for: Characterization of the immune cell landscape of patients with NAFLD
Source: PLoS One. 2020 Mar 13;15(3):e0230307. doi: 10.1371/journal.pone.0230307 (PMC7069622; doi:10.1371/journal.pone.0230307)

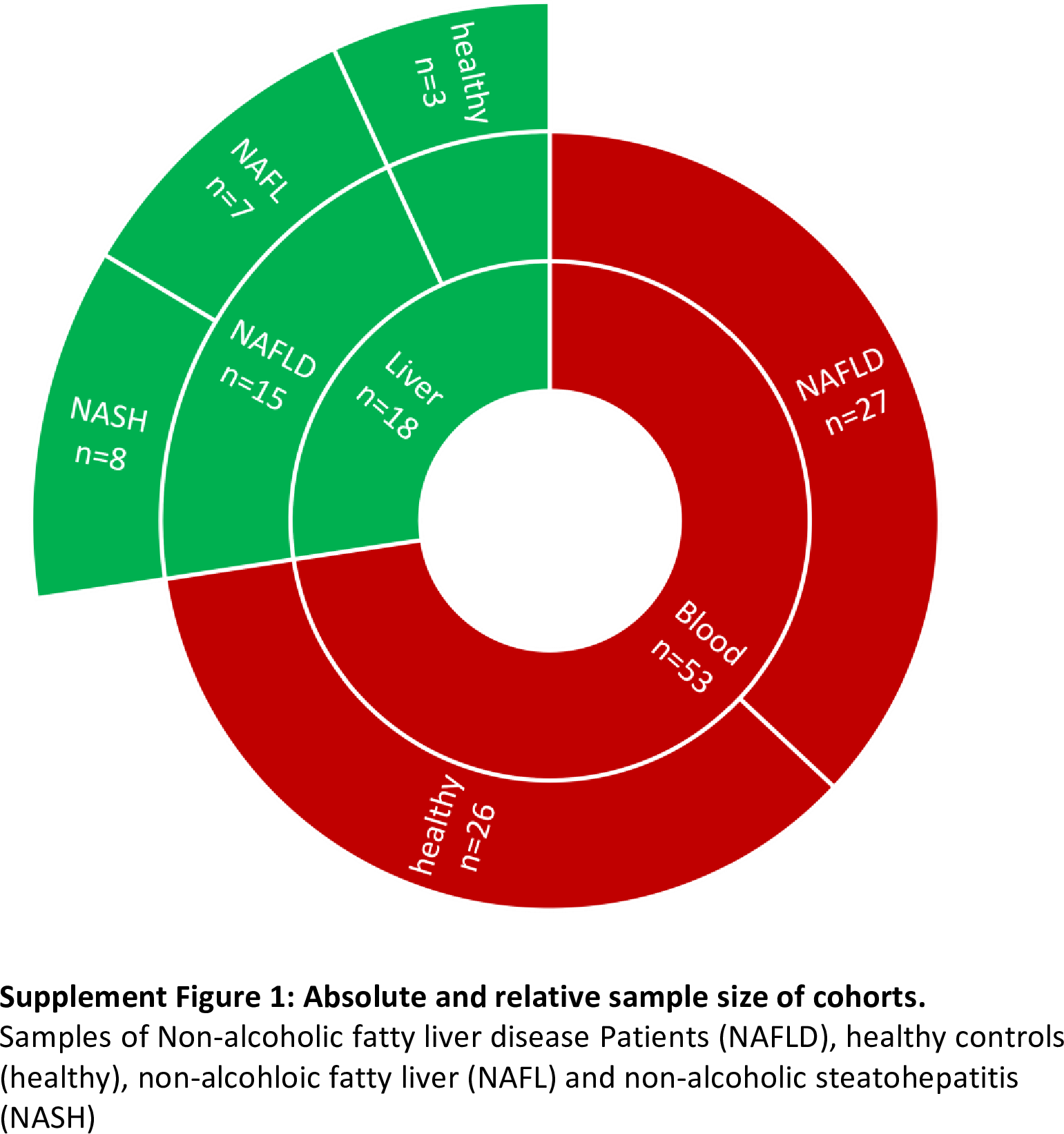

Supplement: S1 Fig — Samples of patients with Non-alcoholic fatty liver disease (NAFLD), healthy controls (healthy), patients with non-alcohloic fatty liver (NAFL) and non-alcoholic steatohepatitis (NASH). (TIF) [file pone.0230307.s001.tif]

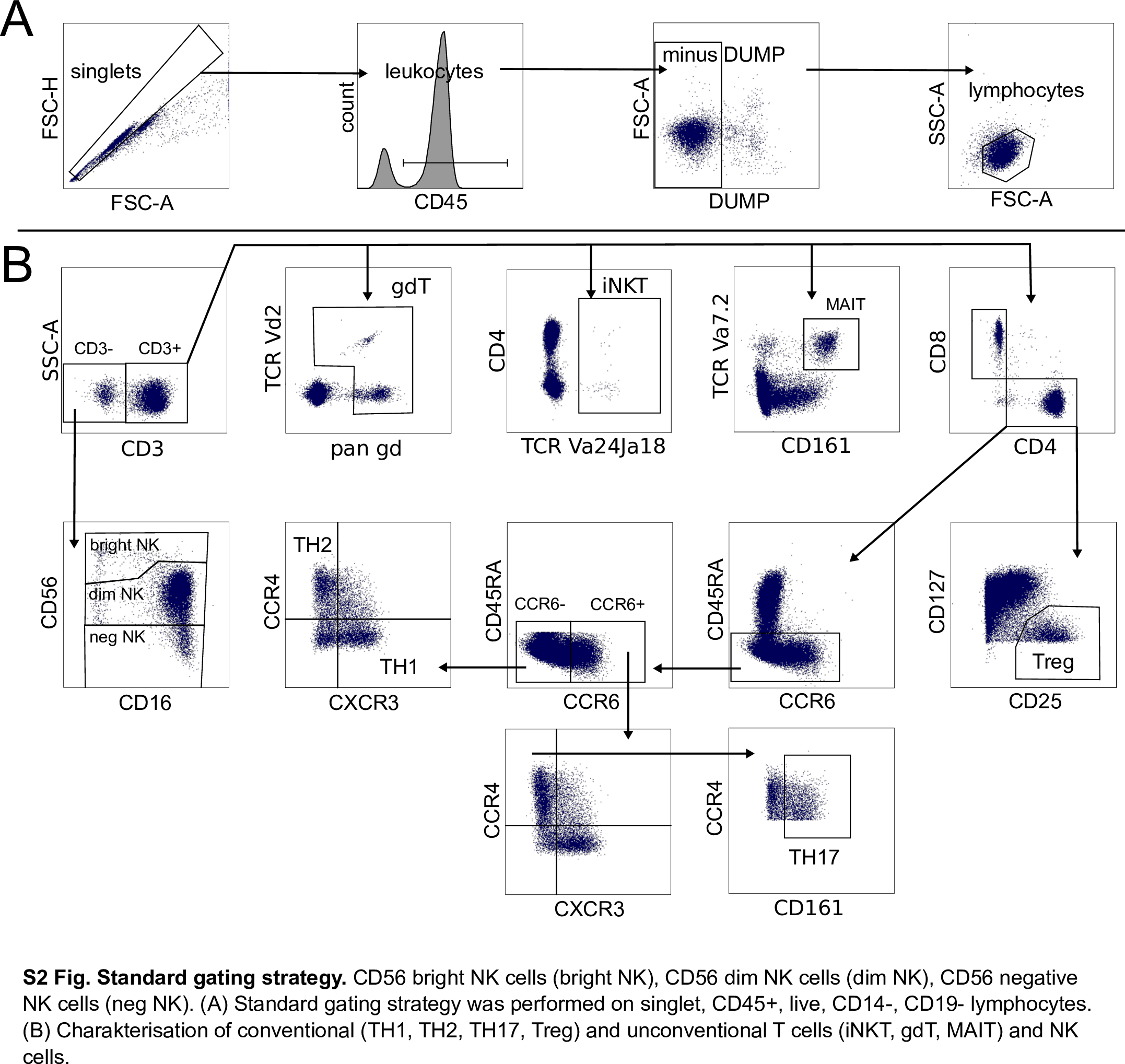

Supplement: S2 Fig — Standard gating strategy was performed on singlet, CD45+, live, CD14-, CD19- lymphocytes. Unconventional T cells and NK cells definition: NKT (TCRVα24Jα18), MAIT (CD161, TCRVα7.2), γδT cells (panγδ, TCRVδ2), NK (CD16, CD56). Conventional T cells definition: T helper cells (CD4+), cytotoxic T cells (CD8), TH1 (CCR6-, CCR4+, CXCR3-), TH2 (CCR6-, CCR4-, CXCR3+), TH17 (CCR6+, CCR4+, CXCR3-, CD161+) as displayed above. (TIF) [file pone.0230307.s002.tif]

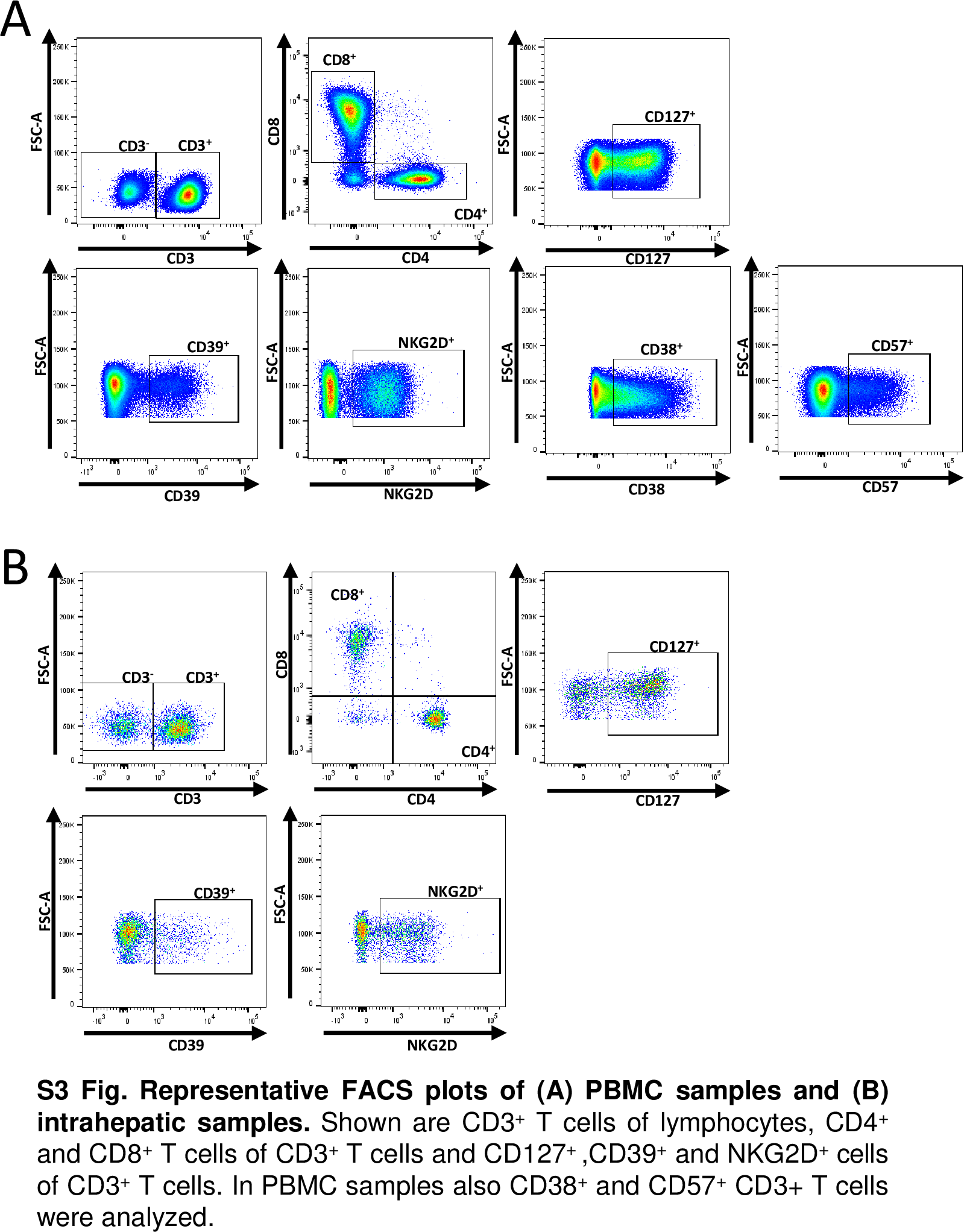

Supplement: S3 Fig — Representative FACS plots of (A) PBMC samples and (B) intrahepatic samples. Shown are CD3+ T cells of lymphocytes, CD4+ and CD8+ T cells of CD3+ T cells and CD127+, CD39+ and NKG2D+ cells of CD3+ T cells. In PBMC samples also CD38+ and CD57+ CD3+ T cells were analyzed. (TIF) [file pone.0230307.s003.tif]

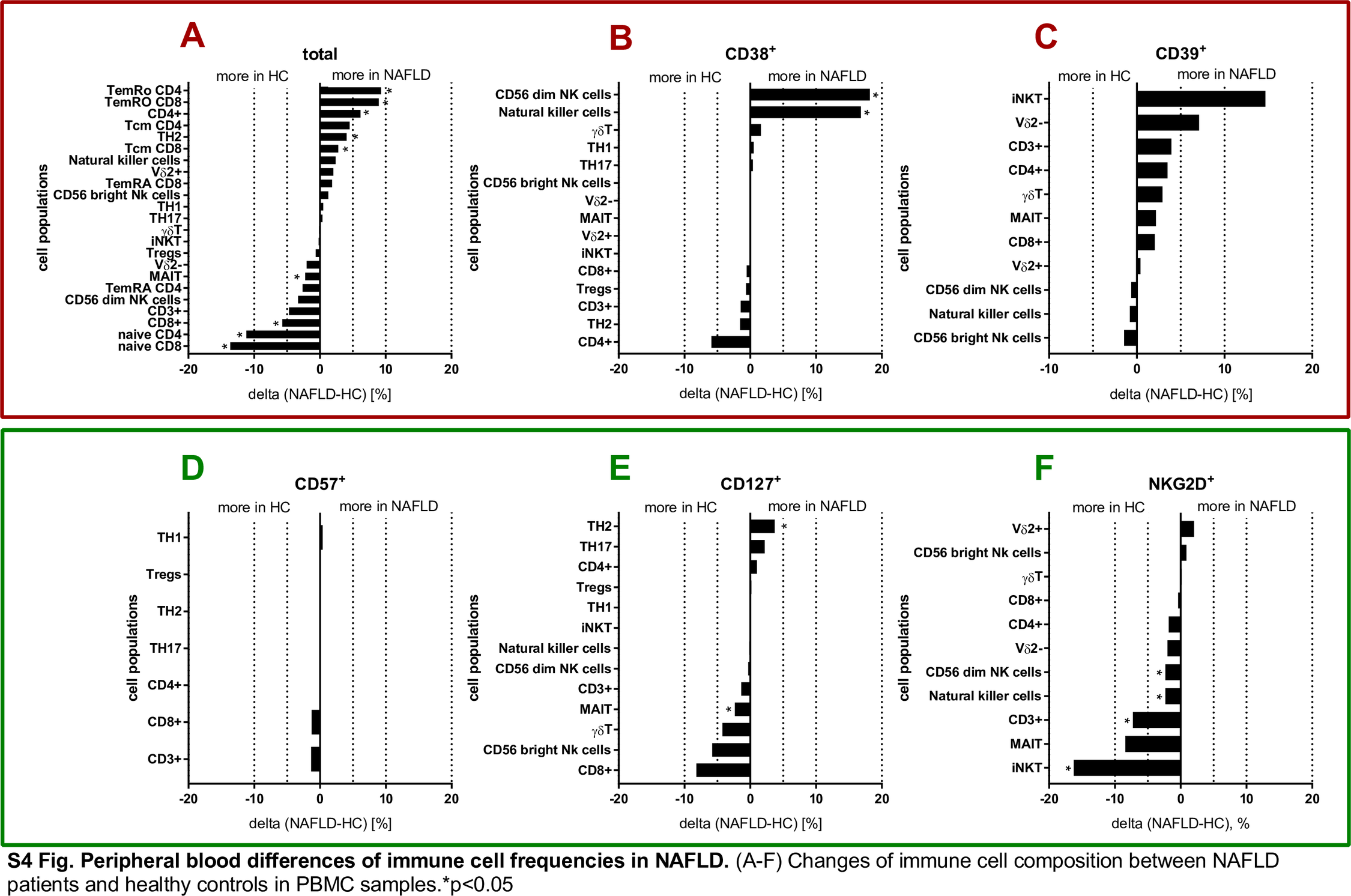

Supplement: S4 Fig — (A-F) Quantified relative differences of immune cell composition between NAFLD patients and healthy controls in PBMC samples. *p<0.05. (TIF) [file pone.0230307.s004.tif]

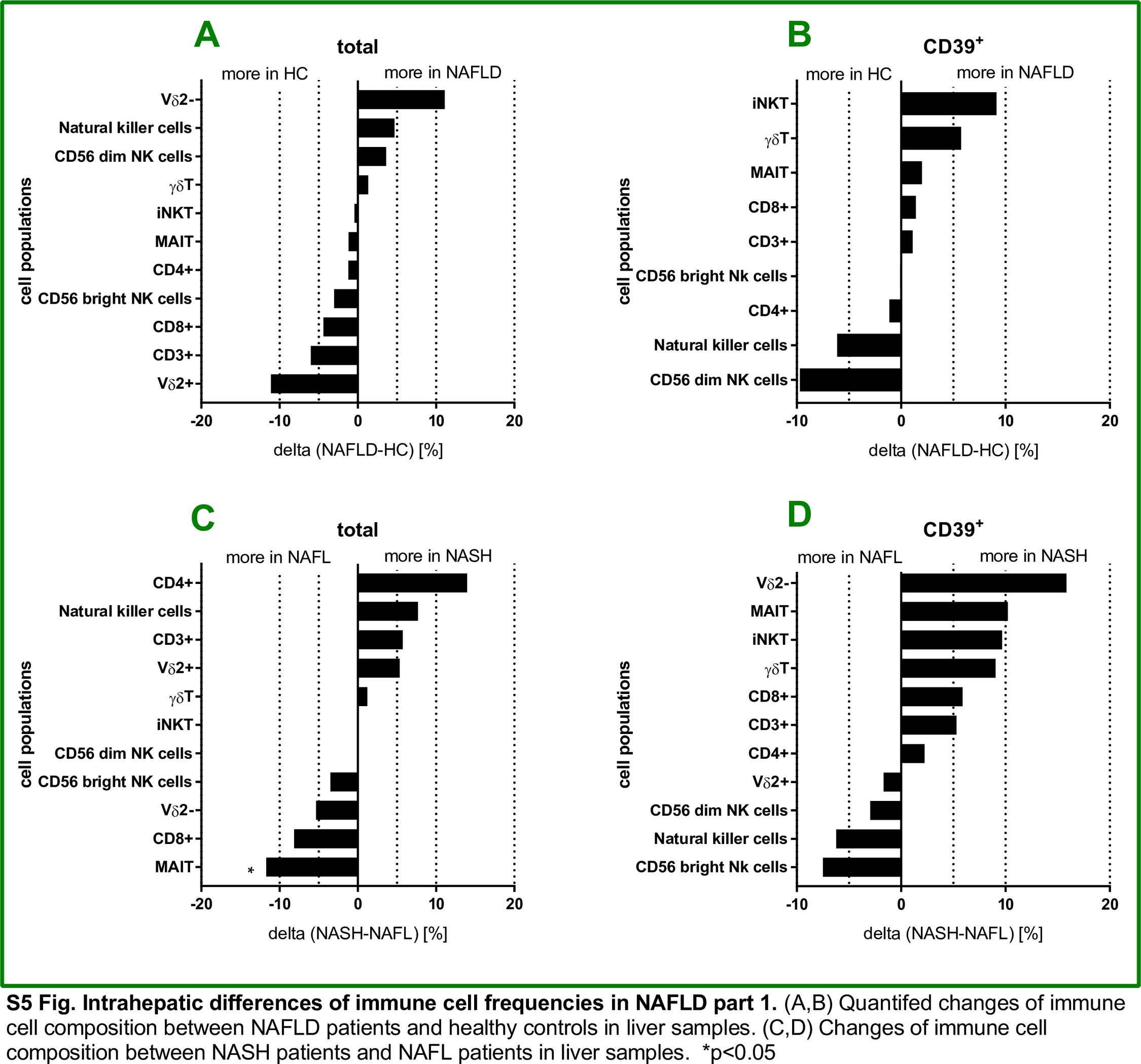

Supplement: S5 Fig — (A,B) Quantified relative differences of immune cell composition between NAFLD patients and healthy controls in liver samples. (C,D) Quantified relative differences of immune cell composition between NASH patients and NAFL patients in liver samples. *p<0.05. (TIF) [file pone.0230307.s005.tif]

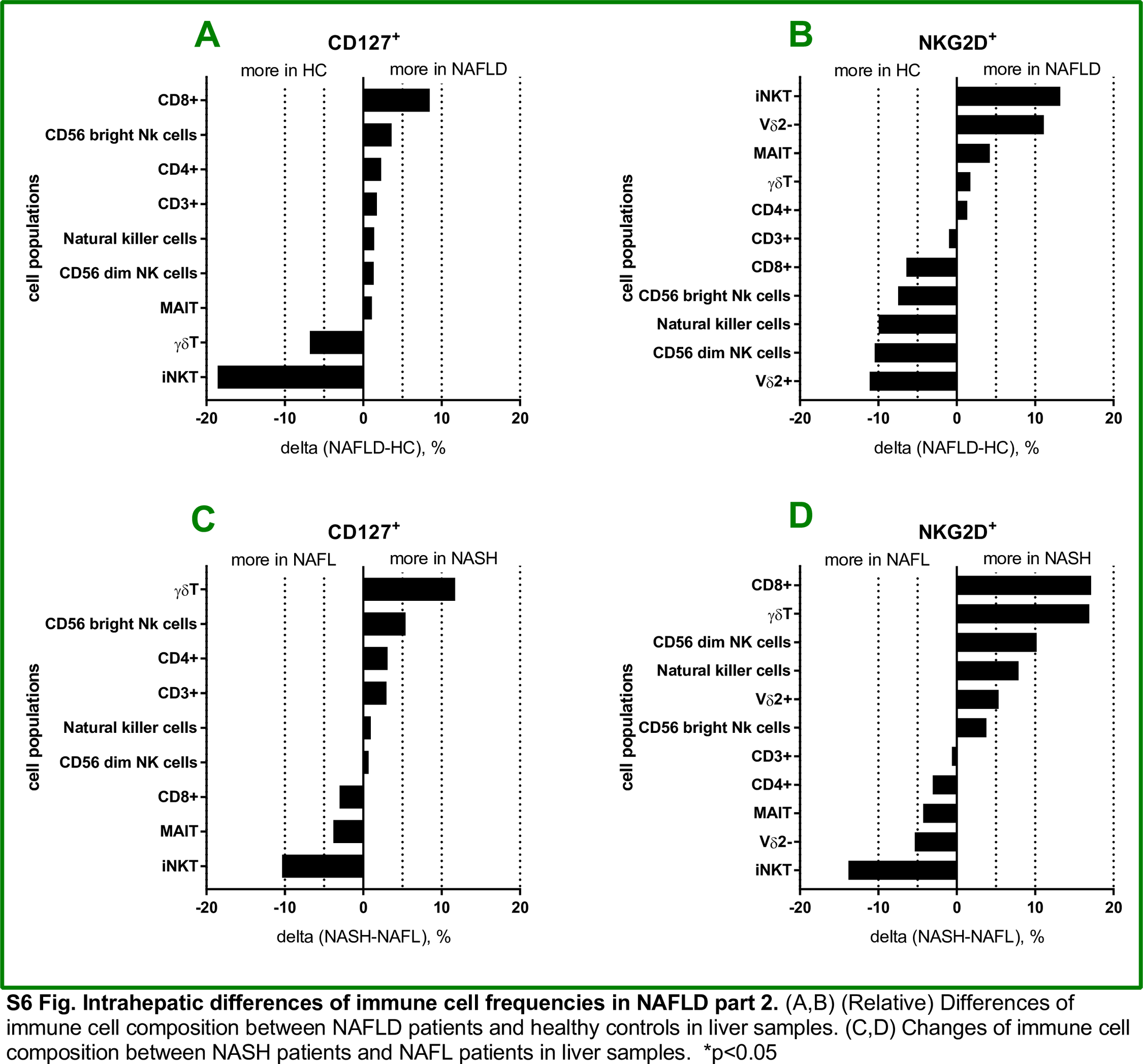

Supplement: S6 Fig — (A,B) Quantified relative differences of immune cell composition between NAFLD patients and healthy controls in liver samples. (C,D) Quantified relative differences of immune cell composition between NASH patients and NAFL patients in liver samples. *p<0.05. (TIF) [file pone.0230307.s006.tif]
