# Supplementary material for: Characterization of the immune cell landscape of patients with NAFLD
Source: PLoS One. 2020 Mar 13;15(3):e0230307. doi: 10.1371/journal.pone.0230307 (PMC7069622; doi:10.1371/journal.pone.0230307)
Supplement: S1 Table — (DOCX) [file pone.0230307.s007.docx]

**S1 Table. FACS panel composition**

| **Panel 1** | | | | |
| --- | --- | --- | --- | --- |
| **Flourochrome** | **Antigen** | **V/sample [µL]** | **Clone** | **Provider** |
| **BUV737** | CD4 | 1 | SK3 | BD Biosciences |
| **BUV395** | CD45 | 0,25 | HI30 | BD Biosciences |
| **BV 785** | CD8 | 0,2 | RPA-T8 | BioLegend |
| **BV 711** | HLA-DR | 0,25 | L243 | BioLegend |
| **BV 650** | CD45RA | 0,25 | HIT100 | BioLegend |
| **BV 605** | CCR6 | 2 | G034E3 | BioLegend |
| **BV 510** | CCR4 | 0,5 | L29144 | BioLegend |
| **BV 421** | CCR-7 | 0,5 | G043H7 | BioLegend |
| **PerCP-Cy5-5** | CD57 | 1 | HNK-1 | BioLegend |
| **FITC** | CXCR3 | 2,2 | G025H7 | BioLegend |
| **PE-Cy7** | CD38 | 0,25 | HIT2 | BioLegend |
| **PE-Dazzle** | CD161 | 1 | HP-3G10 | BioLegend |
| **PE** | CD25 | 0,5 | M-A251 | BioLegend |
| **Alexa Fluor 700** | CD3 | 2 | UCHT1 | BioLegend |
| **APC** | CD127 | 0,5 | A019D5 | BioLegend |
| **APC Cy 7** | CD19 | 0,25 | HIB19 | BioLegend |
| **APC Cy 7** | CD14 | 0,25 | HCD14 | BioLegend |
| **Panel 2** | | | | |
| **Flourochrome** | **Antigen** | **V/sample [µL]** | **Clone** | **Provider** |
| **BUV737** | CD56 | 1 | NCAM16.2 | BD Biosciences |
| **BUV395** | CD45 | 0,25 | HI30 | BD Biosciences |
| **BV 785** | CD8 | 0,2 | RPA-T8 | BioLegend |
| **BV 711** | HLA-DR | 0,25 | L243 | BioLegend |
| **BV 650** | CD16 | 0,5 | 3G8 | BioLegend |
| **BV 605** | TCR Va7.2 | 2 | 3C10 | BioLegend |
| **BV 510** | TCR Vα24Jα18 | 2,2 | 6B11 | BioLegend |
| **BV 421** | NKG2D | 2,5 | 1D11 | BioLegend |
| **PerCP-Cy5-5** | CD4 | 0,1 | SK3 | BioLegend |
| **FITC** | Vδ2 | 0,1 | B6 | BioLegend |
| **PE-Cy7** | CD39 | 2,5 | A1 | BioLegend |
| **PE-Dazzle** | CD161 | 1 | HP-3G10 | BioLegend |
| **PE** | pan γδ | 4 | 11F2 | BD Biosciences |
| **Alexa Fluor 700** | CD3 | 2 | UCHT1 | BioLegend |
| **APC** | CD127 | 0,5 | A019D5 | BioLegend |
| **APC Cy 7** | CD19 | 0,25 | HIB19 | BioLegend |
| **APC Cy 7** | CD14 | 0,25 | HCD14 | BioLegend |
