# Supplementary material for: Characterization of the immune cell landscape of patients with NAFLD
Source: PLoS One. 2020 Mar 13;15(3):e0230307. doi: 10.1371/journal.pone.0230307 (PMC7069622; doi:10.1371/journal.pone.0230307)
Supplement: S2 Table — PBMC, Peripheral Blood Mononuclear Cell. HC, healthy control. NAFLD, non-alcoholic fatty liver disease. HL, healthy liver. Mean immune cell frequencies with standard deviations of PBMC of NALFD patients and healthy controls. p values were calculated with Mann-Whitney test. (DOCX) [file pone.0230307.s008.docx]

**S2 Table. Immune cell frequencies in PBMC of NAFLD patients and healthy controls**

| **PBMC** | NAFLD | HC | p value |
| --- | --- | --- | --- |
| TemRO CD4^+^ cells | 28,32%(SD=11,97) | 19,03% (SD=8,018) | p=0,0107 |
| TemRO CD8^+^ T cells | 25,22% (SD=10,37) | 16,27% (SD=9,624) | p=0,0097 |
| total CD4^+^ T cells | 67,93%(SD=11,83) | 59,9%(SD=7,32) | p=0,0097 |
| T central memory CD4^+^ | 29,72%(SD=9,089) | 25,2% (SD=14,03) | p=0,0633 |
| TH2 cells | 18,41% (SD=6,788) | 14,36% (SD=6,387) | p=0,0417 |
| central memory CD8^+^ T cells | 7,06% (SD=4,91) | 4,261% (SD=4,123) | p=0,0273 |
| naïve CD8^+^ T cells | 29,59% (SD=15,7) | 43,22% (SD=17,5) | p=0,0062 |
| naïve CD4^+^ T cells | 38,39% (SD=14,42) | 49,58% (SD=13,84) | p=0,0060 |
| total CD8+ T cells | 26,12% (SD=11,33) | 32,47% (SD=6,432 | p=0,0173 |
| total CD3^+^ T cells | 78,22% (SD=12,76) | 82,92% (SD=5,794) | p=0,2744 |
| CD56^dim^ NK cells | 84,01% (SD=8,775) | 87,33% (SD=6,444) | p=0,2605 |
| TemRA CD4^+^ T cells | 3,579% (SD=2,54) | 6,202% (SD=11,23) | p=0,2698 |
| MAIT cells | 1,841%(SD=1,738) | 4,073% (SD=3,44) | p=0,0007 |

PBMC, Peripheral Blood Mononuclear Cell. HC, healthy control. NAFLD, non-alcoholic fatty liver disease. HL, healthy liver. Mean immune cell frequencies with standard deviations of PBMC of NALFD patients and healthy controls. p values were calculated with Mann-Whitney test.
