# Supplementary material for: Characterization of the immune cell landscape of patients with NAFLD
Source: PLoS One. 2020 Mar 13;15(3):e0230307. doi: 10.1371/journal.pone.0230307 (PMC7069622; doi:10.1371/journal.pone.0230307)
Supplement: S3 Table — IHL, intrahepatic lymphocyte. NAFLD, non-alcoholic fatty liver disease. HL, healthy liver. Mean immune cell frequencies with standard deviations of IHL of NALFD patients and healthy controls. p values were calculated with Mann-Whitney test. (DOCX) [file pone.0230307.s009.docx]

**S3 Table: Immune cell frequencies in IHL of NAFLD patients and healthy controls**

| **IHL** | NAFLD | HL | p value |
| --- | --- | --- | --- |
| Vδ2^-^ γδT cells | 56,34% (SD=31,1) | 45,23% (SD=26,33) | 0,4412 |
| NK cells | 29,25% (SD=14,37) | 24,57% (SD=13,85) | 0,5098 |
| CD56^dim^ NK cells | 75,77% (SD=12,7) | 72,17% (SD=16,87) | 0,5858 |
| Vδ2^+^ γδT cells | 43,66% (SD=31,1) | 54,77% (SD=26,33) | 0,4975 |
| total T cells | 67,26% (SD=14,95) | 73,27% (SD=14,53) | 0,8273 |
| total CD8^+^ T cells | 39,83% (SD=13,61) | 44,23% (SD=13,2) | 0,4818 |
| CD56^bright^ NK cells | 21,6% (SD=14,15) | 24,63% (SD=18,56 | 0,8235 |

IHL, intrahepatic lymphocyte. NAFLD, non-alcoholic fatty liver disease. HL, healthy liver. Mean immune cell frequencies with standard deviations of IHL of NALFD patients and healthy controls. p values were calculated with Mann-Whitney test.
